# Domain Experts on Dementia-Care Technologies: Mitigating Risk in Design and Implementation

**DOI:** 10.1007/s11948-021-00286-w

**Published:** 2021-02-18

**Authors:** Clara Berridge, George Demiris, Jeffrey Kaye

**Affiliations:** 1 School of Social Work, University of Washington, Seattle, WA USA; 2 School of Nursing and Perelman School of Medicine, University of Pennsylvania, Philadelphia, PA USA; 3 School of Medicine, Oregon Health and Science University, Portland, OR USA

**Keywords:** Dementia, Alzheimer’s disease, Long-term care, Ethics, Decision making

## Abstract

There is an urgent need to learn how to appropriately integrate technologies into dementia care. The aims of this Delphi study were to project which technologies will be most prevalent in dementia care in five years, articulate potential benefits and risks, and identify specific options to mitigate risks. Participants were also asked to identify technologies that are most likely to cause value tensions and thus most warrant a conversation with an older person with mild dementia when families are deciding about their use. Twenty-one interdisciplinary domain experts from academia and industry in aging and technology in the U.S. and Canada participated in a two-round online survey using the Delphi approach with an 84% response rate and no attrition between rounds. Rankings were analyzed using frequency counts and written-in responses were thematically analyzed. Twelve technology categories were identified along with a detailed list of risks and benefits for each. Suggestions to mitigate the most commonly raised risks are categorized as follows: intervene during design, make specific technical choices, build in choice and control, require data transparency, place restrictions on data use and ensure security, enable informed consent, and proactively educate users. This study provides information that is needed to navigate person-centered technology use in dementia care. The specific recommendations participants offered are relevant to designers, clinicians, researchers, ethicists, and policy makers and require proactive engagement from design through implementation.

## Introduction

Investment in technology to support dementia care is growing as demand for human and financial resources to support care outpaces its supply (AARP and National Alliance for Caregiving 2020). The functions of technological tools range widely, including activities of daily living (ADL) assistance, behavioral and health monitoring, cognitive assistance and monitoring, and environmental and emotional support (Choi et al. 2019; Seelye et al. 2020; Piau et al. 2019; Czaja et al. 2019; Orlov 2019). While ethical implications of the use of technologies for people living with dementia have been slow to come into focus (Ienca et al. 2018; Robillard et al. 2018; Novitzky et al. 2015), issues such as autonomy, informed consent with dementia, dignity, and distributive justice, along with threats to values like privacy and identity, are beginning to receive heightened attention in the literature (Meiland et al. 2017; Robillard et al. 2018; Mulvenna et al. 2017; Sánchez et al. 2017; Berridge 2016; Boise et al. 2013). Researchers have identified adoption barriers and user dissatisfaction that result when ethics and implicated values are not engaged in the development of devices (Robillard et al. 2018). The surveilling nature of some of these technologies can also put important values—and family members themselves—in tension with one another (Kenner 2008; Berridge and Wetle 2019).

An expert study that probed some of the pressing issues with Internet-connected technologies in dementia care was recently conducted in Italy, Switzerland, and Germany (Wangmo et al. 2019). Twenty medical workers, nursing home managers, and researchers in geriatrics and related fields were asked about ethical concerns with the use of intelligent assistive technology with older adults and people living with dementia. A range of concerns were described, including fair technology access, the possible replacement of human assistance, and the use of deception. The ethical importance of communicating risks and benefits to users of a given technology and consenting patients were core concerns (Wangmo et al. 2019). These concerns are especially amplified in this period of rapid technological development, with marked growth of innovations in robotics, artificial intelligence (AI), sensor-based systems (Vallor 2016; Lindeman et al. 2020), including in-home sensors that monitor movement and behavior, and voice-activated systems that access information or remotely control appliances. A recent systematic review found that most (67%) dementia care technologies have been developed without explicit consideration of any ethical principles (Ienca et al. 2018).

What information is needed to prepare to navigate new technology-based practices in home-based dementia care in an way that respects the values that matter to individuals and families? One need is to be able to project as best we can what technologies to focus on. Another need—underscored by the ethics research—is a clear understanding of their potential risks and benefits. While big-picture ethical considerations must come into sharper focus (including questions about access, autonomy, and what constitutes good care), we must also examine the specificity of individual technologies and their attendant uses and risks (Pols 2017).

This study attends to these needs by consulting transdisciplinary aging and technology experts from academia and industry. The goal is threefold: to learn which technologies are predicted to be the most prevalent in five years; to understand the potential benefits and risks of each technology; and to identify options to mitigate those risks. Our hope is that these findings will allow for a concrete assessment of ways to address the risks and value tensions that technologies used by families in the home create. For this applied approach, we focus on identifying and learning about those technologies that are the most likely to be threatening to values that older adults may care about (i.e. privacy, independence) and thus most warrant a conversation with an older patient with mild dementia when families are deciding about their use. The overarching aim is to learn where to focus efforts for the near-term and to proactively foster technology-mediated practices in the home that families can feel good about.

## Methods

### Participants and Recruitment

Potential participants were identified in two ways. First, the three authors who are disciplinarily dispersed within the field of gerontechnology collectively identified an initial list of experts to invite. We sought a balanced mix of people with specific expertise in technology for older adults from industry, AI, and gerontology and related social sciences, including ethics. Second, some of these industry contacts and leaders in gerontechnology—including nonparticipants—were asked to help identify potential participants and those with specialized knowledge in under-represented areas of AI or ethics. Contacts were also asked if they could recommend others to invite from their own respective fields. The combined process yielded a list of 37 potential participants.

Participants were domain experts in aging and technology research, design, or implementation in the U.S. and Canada who had been in the field for more than five years. A criterion for academics was that they had published at least five peer-reviewed articles on technology and aging; those in industry had recent work experience with technology products for elder care.

The sample size for the Delphi approach we pursued is not a statistically bound decision (Akins et al. 2005; Ziglio 1996), and 15–20 respondents is the typical range (Hsu and Sandford 2007). A pre-Delphi invitation to participate in the process was first sent to the 37 domain experts (Sinha et al. 2011). Each received one email invitation, and those with away messages received two. Participants were informed that this study would be the first step of a larger project of developing a new communication and education tool for early-stage dementia family-care partners to discuss how technologies may be used in their care. The role of this Delphi process in the development of the tool was to help determine which technologies should be featured in the tool in order to make it as relevant over time as possible.

Twenty-five responded that they were willing to participate in the study; this rate of agreement to participate of 67.5% was expected based on the Delphi literature (Mokkink et al. 2010). These 25 received the Round 1 survey and 21 of them completed it. There was no attrition between the two rounds: 21 participants completed Round 1, and all 21 completed Round 2, for an 84% response rate (21/25). Of these participants, 15 (71%) worked primarily as academics, many of whom had experience developing technology; and 5 (24%) represented industry. One (5%) was a consultant and industry analyst in the aging and technology space. Participants are listed in the Appendix.

### The Delphi Process and Analysis

The Delphi technique is used to solicit experts’ opinions and to build consensus (Hsu and Sandford 2007). To begin this process, a Round 1 structured questionnaire was distributed with nine questions. Participants were presented with nine technologies (e.g., virtual reality) and data types that monitoring technologies capture (e.g., location outside of the home) based on an extensive review of the literature. Since device forms will change more rapidly than will the types of data collected, the focus was on generic technologies or data types rather than specific products. Participants were asked to describe the primary benefits and risks for each for use in the care of a person with mild dementia. After listing the potential risks and benefits for each provided technology, participants were asked to expand and comment on the technology categories. Beginning a two-round survey with a Round 1 structured questionnaire is an acceptable modification of the three-round Delphi approach that begins with an open-ended questionnaire (Hsu and Sandford 2007). The Delphi method requires a minimum of two rounds (three if round one is open-ended) (Walker and Selfe 1996). As Walker and Selfe point out “repeated rounds may lead to fatigue by respondents and increased attrition” (p. 679). Considering the challenge of attrition for our expert participants, we decided to pursue a two-round approach.

For Round 2, twelve technology categories were rearticulated based on the most commonly reported beneficial uses for each and suggestions for missing categories from Round 1. Based on Round 1 responses, descriptions of technology purposes were refined, one category was subdivided into two, and two new technology categories were added: augmented reality and smart homes for environmental control.

The refined technology descriptions were presented for participants to rank according to those that they perceived would be most commonly used in the care of people with dementia in five years. Participants were then asked to select among the twelve technologies the ones that most warranted a conversation between an older person with dementia and the family members who were deciding about using them. Participants were prompted to consider which were the most likely to be threatening to values. Each question was followed by a prompt for participants to describe their rationale for their selection. Ranking and selecting questions had answer choices that were shuffled for each participant.

The most commonly noted risks generated in Round 1 were extracted and reported back to participants in aggregate in Round 2. Participant responses were anonymous to other participants. This report-back was followed by a question to identify design options that families might choose to mitigate the risks of using technologies in care. Participants were asked to describe realistic design options to mitigate as many of the noted risks as they could think of.

Rankings were analyzed using frequency counts. Written-in responses were thematically analyzed using an inductive, reflexive thematic analysis approach by the first author and then validated by another member of the team (Clarke et al. 2019). Participants were compensated $300 for completion of the two surveys. This research was approved by the University of Washington Human Subjects Division.

## Results

### Near-Future Predicted Use

After participants commented on the selection of technologies in Round 1, they were presented in Round 2 with a refined list of 12 technology categories, which are presented in Table 1.

**Table 1:** Twelve refined categories of technologies used in dementia care

Smart home systems to control environmental settings and appliances	
Augmented reality to give the person with dementia digital cues to support home environment and task navigation	
Virtual reality for cognitive stimulation, relaxation, reminiscence therapy, behavior management	
Location tracking within the home to detect variation in patterns of activity	
Location tracking outside of the home to monitor safety and social activity	
Detecting movements through apartment door to confirm return home and monitor outdoor activity frequency or presence of workers, meal delivery, visitors	
Video conferencing that allows a caregiver to turn the webcam on and "enter" the room visually for social connection and visual assessment of person and home	
Remotely monitoring physiological variables for frequent assessment to predict and manage health-related risks	
Recording audio in a person’s home to respond to emergencies and security threats, monitor socialization, or detect cognitive change and other conditions	
Using an AI conversational agent to stimulate engagement and connection with the outside world (accessing music, information, web-conferencing with people)	
Using an AI virtual agent or socially assistive robot for non-human companionship to ease loneliness and prompt cognitive or physical engagement	
Using a robotic assistant to help with physical aspects of daily living	

They were then asked to rank according to those that would be the most prevalent in dementia care in five years and to explain their rankings. Prediction proved difficult. While the technologies that would be most common were ranked fairly consistently, there was no agreement about how near they were to widespread use. Projections varied widely, from “Some will be obsolete by five years” to “I believe all of the above will be very prevalent” and “I think widespread use of all these are more than five years away-more like ten.” One participant explained, “We generally over estimate what is possible in two years and underestimate what is possible in five years. In many of these examples, we are assessing technical capacity (which is high in 5 years) with willingness to adopt (which I think is low).” Others observed that the gap between cost and income of those caring for older adults will limit uptake.

Without reaching consensus about realistic timelines, participants on the whole believed that the following would be the most prevalent in five years (most to least frequently endorsed):

1. Smart home systems to control environmental settings and appliances
2. Video conferencing that allows a caregiver to turn the webcam on and “enter” the room visually for social connection and visual assessment of person and home
3. Location tracking outside of the home to monitor safety and social activity
4. Location tracking within the home to detect variation in patterns of activity
5. Detecting movements through apartment door to confirm return home and monitor outdoor activity frequency or presence of workers, meal delivery, visitors
6. Remotely monitoring physiological variables for frequent assessment to predict and manage health-related risks
7. Using an AI conversational agent to stimulate engagement and connection with the outside world (accessing music, information, web-conferencing with people)
8. Recording audio in a person’s home to respond to emergencies and security threats, monitor socialization, or detect cognitive change and other conditions
9. Using an AI virtual agent or socially assistive robot for non-human companionship to ease loneliness and prompt cognitive or physical engagement

Key insights were that those technologies that already exist for a wider market, such as free GPS software on phones, smart home systems, and activity monitors, were predicted to be more prevalent sooner than those specifically targeting older adults. Those that are becoming ubiquitous were poised to be readily adopted for care purposes. With the growing ubiquity of smart speakers also comes greater attention to failures of data security, as one participant suggested: “Audio recording will be delayed in the consumer market, in part due to the strongly negative reactions we're seeing to things like smart speakers recording audio (e.g., bad reactions to audio recording in Amazon's Echo and Google's Home products).”

### Technology Agnostic Potential Benefits and Risks

When the potential benefits and risks associated with each technology were identified (see Table 2), some of these were found to be technology independent, common across most of these individual technology categories. Concern over the security of audio and other data types was one of these common issues. Participants emphasized both security risks and nefarious use as unresolved problems. The issues were raised of data exfiltration by hackers or Internet service providers and “Big data collection needed without much knowledge for where it may end up.” Much of the data collected by these technologies can be accessed by third parties, which leads to privacy risks for anyone whose data might be captured, including visitors to a home where the technology is in use. Participants voiced concern that recordings that lead to knowledge of non-adherence or high-risk activities could in turn lead to increased premiums or denial of healthcare coverage.

**Table 2:** Risks and benefits by technology or data type

Technology category	Primary potential benefits	Primary potential risks
**Smart home systems**	Remotely control appliances, lighting, home security, and thermostats Detect deviation from routine based on device input Enable independence	Data security and nefarious use Privacy invasion Requires back-up generators to power during natural disasters and rolling blackouts
**Video conferencing that allows a caregiver to turn the webcam on and “enter” the room visually**	Social connection in context of caregiver shortage Visual assessment of person, affect, home conditions, medication and meal oversight Ability to see ADL/IADL needs Monitor visitors Provide real-time coaching or support Avoid need for travel and associated risks	Substitute for/reduction of personal visits or calls Unease with knowledge that caregiver could enter at any time and inability to know when alone Create suspicion/distrust/paranoia/anxiety Sudden and unexpected visual presence is jarring Extreme violation of personal privacy if no ability to accept Bedrooms/sexual desires/reveal things person would choose not to share Privacy invasion for visitors Loss of independence Self-limiting activity Caregiver could unnecessarily harass Face recognition can be deployed, risking privacy
**Location tracking outside of or within the home**	Monitor safety and social activity Locate wandering or lost individuals Detect a possible fall Verify sleep, eating, activity habits and assess patterns for early intervention without questioning the individual Provide basis for prompting (eating or going to bed)	Lost sense of privacy in one’s home Feel there is no place to hide/uncomfortable in home Infantilizing or feeling “baby sat” Lead children to be too intrusive, increase friction Autonomy limited if used to control activities in response to perception of sedentary behavior Unnecessary pathologization of departures from routine Caregiver stress with ambiguous data/alert overload Reduce social calls, transforming routines to task-oriented
**Detecting movements through apartment door**	Confirm return home and monitor outdoor activity Monitor arrival and departures of workers, meal delivery and visitors Identifying patterns that may raise questions of safety or social isolation Prevent a wandering incident	Autonomy breach/behavior change when being observed to conform with expectations Lead to intrusive questioning by caregivers/paternalism Unnecessary pathologization of departures from routine False positives and unspecific data Unnecessary intervention that causes agitation or confusion Potential over-reliance on system Privacy invasion
**Remotely monitoring physiological variables**	Provides hard-to-collect data like sleep data Predict health-related risks Early identification of illness Detect changes in real time and trigger alerts or emergency services Manage health-related risks through frequent assessment over time, particularly cardiac arrhythmia and other detectable medical events Data can be shared with medical professionals Fewer caregivers can monitor and serve more patients Reduce clinic/home health visits for basic vital checks Could indicate neglect of an important routine (e.g., getting close to severe dehydration)	Overtreatment or unnecessary medical intervention Cause worry or create a mindset that “I'm at risk for sudden death” Data overload; difficult to interpret/make actionable False sense of security that medical issues will be detected More medication or polypharmacy based on wrong application by people without the proper knowledge Privacy invasion; feeling of "being monitored" Not wanting caregiver to know how sleeping or with whom Restriction of behaviors because of monitoring (caregiver uses data to rebuke, restrict, or micromanage, limiting autonomy)
**Recording audio in a person’s home**	Emergency alerts (falls or other need for help) Security alerts (intruder or abuse) Monitor socialization Speech analysis to detect cognitive change, disease progression, depression, and auditory hallucinations Enhance understanding about how person moves throughout home and their activities Monitor workers or presence of others in the house	Privacy invasion if no ability to accept Privacy invasion for visitors Compromise dignity when sexual/bodily functions captured Threats to agency and autonomy Feeling of being watched at all times Fear, paranoia, and depression when a person cannot be sure that their home not their domain to control Feeling uncomfortable/bugged/no place to hide Information overload and who will do the monitoring? Overreacting caregivers/What warrants intervention? Overreliance compromising proactive help Wiretapping law and liability based on caregiver in/action?
**Using an AI virtual agent or socially assistive robot**	Provide information (health, weather, etc.) Prompt cognitive or physical engagement (entertainment, music, AI conversation) Reminders (medications, appointments) Home shopping Facilitate calls to people for social connection Collect self-report data for assessing health, cognition, well-being Voice control of home systems promote independence Opportunity for person to use their language functions	Errors that cause frustration for people with dementia Accidental unwanted actions (purchases, appliances off) Deception or confusion about who is behind the AI Confusion about where voice is coming from Make things feel strange Interrupting concentration and tasks, causing fear/confusion Give false information, dangerous if acted on (health)
**Using an AI conversational agent**	Non-human companionship to ease loneliness Enable auto check-ins Monitoring capability with cameras and microphones Use conversation data to detect cognitive changes Collect self-report data for assessing health, cognition, well-being Opportunity for person to use their language functions	Reduced human interaction, increased isolation Deprive people of meaningful conversations Depersonalization in robot relationships (poorly understood) Errors cause frustration for people with dementia Possible deception or confusion about who is behind AI Overreliance Privacy violation if records conversations (likely) Confusion about where voice is coming from Misleading marketing materials imply that use can "roll back" symptoms of dementia, shaping expectations
**Using a robotic assistant**	Help with physical aspects of daily living Assist to an upright position Assist with housekeeping to enable independent living Preserve dignity in intimate ADL care Accompany to reduce fall risk Provide intellectual and physical challenges Provide company and increase emotional well-being Provide reminders Augment human staffing shortages Reducing risk of caregiver injury Caregiver could move the robot around the home to see things	Privacy invasion Overreliance for daily tasks and increase speed of decline Reduce human visits/contact and human help Contribute to isolation and loneliness Malfunction/operation challenges and maintenance Risks to physical safety Confusion/disorientation/fall risk Lack of emotional alignment causing distress Perceived as dehumanizing If networked into a larger system, its cameras and audio system may be able to be commandeered and relayed to a third party Face recognition can be deployed, risking privacy
**Augmented reality**	Digital cues to support home and task navigation through real time, digital information about any object in the real world (reminding how to take medicine, use a medical device, giving dementia friendly instructions) Skills training by superimposing virtual instructions Support memory related problems (people and daily task recall) Presence or remote cuing of caregiver	Information is limited to prepared scenarios Lack of flexibility and assessment of users’ performance Cause confusion or disorientation
**Virtual reality**	Behavior management (such as sundowning) Memory practice Reminiscence therapy Cognitive stimulation Relaxation Pain management Entertainment, gaming, and adventure experiences Assistance with caregiver empathy training	Challenges in transition from VR to the real world Need to manage devices, cleanliness, and content updates Loss of interest over time Cause one to avoid staying active or engaged socially Confusion and agitation, disorientating Cybersickness Vertigo and disorientation could create a falls hazard Requires visual acuity Flow state of caregivers may cause them to neglect person Data are typically monitored by the vendor—privacy is threatened by data breaches or intentional monitoring

Prolonging independent living and caregiver peace of mind were seen as potential benefits across all technologies, while unactionable data, information or alert overload, and caregiver fatigue were cited as likely to make caregivers anxious. Other commonalities across technologies included risks of inaccurate data, false positives and negatives, and a false sense of safety. Finally, the problem that all Internet-connected devices require access to the Internet was identified as creating an impediment to use.

Other potential benefits and risks are specific to a given technology. The summarized potential benefits and risks of each individual technology is provided in Table 2.

### Mitigating Risks

Participants were then presented with the aggregated risks most commonly noted in Round 1 (shown in Table 3) and asked in Round 2 to identify options to mitigate the risks of using these technologies in care.

**Table 3:** Aggregated predominant risks of technology use

Privacy invasion for visitors and person living with dementia (reveal a state that an individual finds embarrassing or would choose not to share)	
Infantilizing or feeling “baby sat”	
Feeling uncomfortable/bugged/no place to hide, leading to fear, paranoia, depression	
Pathologizing departures from routine	
Caregiver stress from ambiguous data or alert overload	
Reduction of personal visits or calls, leading to loneliness or isolation	
Overtreatment or unnecessary intervention	
False sense of security that issues will be detected, reducing proactive help	
Tension caused by caregiver paternalism in form of intrusive/harassing questioning	
Caregiver limits freedom, using data to rebuke, restrict, or micromanage	
Care recipient changes behavior to conform with expectations	
Unease with knowledge that caregiver could enter the room at any time and inability to know for sure when alone, creating suspicion/distrust/paranoia/anxiety	
Over reliance on tech for daily tasks, speeding decline in functional capacity	
Lack of emotional alignment causing distress	
Compromising dignity (bodily functions and sexual activities captured)	
Data lead to knowledge of non-adherence or high risk activities that lead to increased premiums or denial of coverage	
Data security risk leading potentially to nefarious use	
Unknown or unsanctioned third party use	
Problems with errors, causing frustration for people with dementia	
Person with dementia accidentally triggers unwanted actions	
Possible deception or confusion about who is behind an AI agent or robot	

Participants acknowledged the great difficulty of addressing these challenges. One wrote, “This is really hard, all the concerns are very valid.” Another participant responded:

Wow this is a tough one. I believe that technology is best considered as a way to better enable or drive efficiencies with the in-person caregiving function rather than serve as a replacement for human touch/interaction. I feel that when considering the use of technology to enhance the care of adults with Alzheimer's or dementia it becomes even more critical to consider how the technology will primarily assist the caregiver and reduce their burden rather than be used as a substitute (which can contribute to so many of the unwanted outcomes identified in the above list) for their in-person engagement.

One participant explained the importance of getting implementation right:

The issue is not so much design options for the tech itself (although important), but ensuring that technology - whatever it is - is applied in the right way (e.g., care professionals have the right care process in place, including consent, services are appropriately regulated, tech outcomes are evaluated, consumers are sufficiently “educated” to understand the benefits and costs, etc.).

Suggestions given by participants for risk mitigation are outlined below and summarized in Table 4. They are categorized as follows: intervene during design; make specific technical choices; build in choice and control; place restrictions on data use and ensure security; require data transparency; enable informed consent; and proactively educate users.

**Table 4:** Ways to mitigate risks

**Intervene during design**	
Employ existing frameworks	
Value sensitive design	
Privacy-by-design framework	
Ethical adoption framework	
Convene multidisciplinary design teams with expertise in cognitive impairment, ethics, and product testing	
Prior to deployment, conduct end-user and usability testing	
Prior to deployment, conduct ethics studies	
Do not design AI devices to substitute for human interaction	
Avoid humanoid design and deception	
**Technical choices**	
Use non-identifying, privacy enhancing methods (sensor before camera; silhouette before image)	
Uncouple data sources	
Prohibit extraneous data collection	
System check-in with individual before alert is triggered	
Graded alerts	
Auto-tag and remove activities compromising the person’s dignity	
Avoid continuous streaming—require permissions for video or audio	
Enable two-way feed with caregiver	
Titrate level of support to need to prevent skill loss	
**Build in choice and control**	
Enable ability for PWD to pause system/data collection	
Enable data deletion and approval by individual	
Employ end-user defined function metrics	
Allow PWD to stop or not use	
Provide alternative options to enable actual choice about use	
**Restrictions for data security and unsanctioned third-party use**	
Mandatory data security practices: two-factor authentication, encryption, HIPAA compliance, permissions	
Individual or family owns their data	
Disclosures that data won’t result in higher health insurance costs	
Ensure that enrollment is NOT through healthcare or insurance related benefit	
Data should not be used for marketing	
Require informed consent for use other than care provision, including research	
Guaranteed assurances of data privacy	
**Require data transparency**	
Regarding: where data are, who can access, what are the algorithms to trigger intervention, possible data monetization	
Devices actively remind users of how they operate, who is controlling them, and how data are being collected and used	
**Enable informed consent**	
PWD should be counselled about the risks and benefits associated with monitoring, use of data by whom	
Establish informed consent as a process	
Repetitive opportunities to try it out, decline to use, and try again	
Advanced directives for technologies that monitor and use AI	
Inclusion of PWD in installation and onboarding process	
**Proactively educate users**	
Disclosures	
Data may need clinician interpretation	
Oversight and backup provisions are necessary	
AI is not a substitute for contact with a person living with dementia	
User manuals	
Mandatory onboarding materials	
Service wrap-around	

#### Intervene During Design

Participants suggested three specific approaches that designers and engineers could use to inform development of dementia care technologies: value sensitive design, the privacy-by-design framework, and the ethical adoption framework. These recommended frameworks are described in the discussion section. Other suggestions that are compatible with these frameworks were to convene multidisciplinary design teams; to engage gerontechnologists with expertise in cognitive impairment, ethics, and product testing; and to conduct careful end-user testing, usability, and ethics studies prior to deployment. Ethics studies, which could be done quickly and inexpensively, were described as critical.

One particular design-stage ethical concern was raised repeatedly. Participants worried that some of the technologies, particularly those that are AI-driven, could be used to justify or enable reduction of human contact. One wrote, “Overall I continue to be very concerned about the ethics and invasiveness of some tech. AI and use in loneliness has little data and I worry about attempting to substitute real human connection.” Another explained,

An AI for non-human companionship is problematic because it undermines perceived connection to family/care. Although family/care may not be any less, the introduction of the non-human companionship can be perceived as replacing family/care. AI to stimulate engagement does not have this same problem. It's about addressing boredom, encouraging mental stimulation, not about companionship.

This was a common distinction made between appropriate and potentially inappropriate uses of AI. Some cautioned that substituting AI for human contact could also speed decline in functionality, emphasizing the importance of avoiding technology that “substitutes for maintaining personhood.” Participants noted that humanoid design should be avoided to reduce the risk of deception around AI.

Participants also spoke to the difficulty of mitigating all the risks when much depends on the implementation of the technologies: “It’s hard to control for the dynamics of interpersonal/family relationships. If a product is designed to do something that invades privacy and trades off that for safety, then there will always be this issue.”

#### Technical Choices

Participants suggested specific technical options that could be designed in. One was that non-identifying methods should be used whenever possible. For example, while a camera could be used to detect falls, heat sensors or smart floors would preserve privacy more effectively. Participants wrote about the need to embed privacy preserving features in surveillance technologies, such as uncoupling data sources, capturing silhouettes rather than actual images, and collecting only necessary data while balancing between utility and data granularity.

To prevent triggering unwanted actions, participants suggested creating a system of checks and balances such that the system checks in with the person to gauge status before sending an alert. Alerts could also be graded to specify certainty that a negative event had occurred. Computer vision and machine-learning algorithms could be used to tag and remove activities that are likely to compromise the person’s dignity. Participants suggested requiring permissions for intrusive applications, including video or audio, instead of continuous streaming. In an effort to equalize power relations between a person living with dementia and a caregiver, two-way feed could be provided when using audio or video capture.

To mitigate over-reliance on technology, systems could require people to exert some form of physical effort in the accomplishment of a task. One participant noted that “to reduce risk of over-compensation and associated skill loss and impact on independence and identity, technology could be engineered in a ‘reverse training wheels’ model (i.e., start with minimal and elder-controlled settings, with escalation of services based on severity of dementia).”

#### Build in Choice and Control

Nearly all participants emphasized the need for control to be offered to older adults and their families in the design and use of these technologies. One participant underscored the difficulty with generalizing preferences to the population category “older adults,” writing that “responses to a given technology will depend on many personal, social, and situational factors.” They continued, “Generalization is difficult because you need to identify which values are important to the older adult. For some it’s safety, others privacy, independence, personal control. Preference affects response to and usage of technology.” In concert with this important point, participants agreed that designers should adhere to the core design principle of providing personal control options for any system that monitors individual activities. One explained the importance of enabling a pause option: “My prior work found that allowing the older adult to pause whatever the technology is helped with their concerns. They rarely paused it, but being able to was psychologically calming.”

An additional specific way to give consumers control is allowing them to delete data and approve data before sharing it in non-emergency situations. One participant suggested setting limits on the capabilities of the technology to remain within the bounds that a person understands and can control. Yet another innovative approach is to use end-user defined function metrics as goals rather than physiological metrics when deciding alert thresholds.

Finally, while it may be apparent to those unfamiliar with familial care dynamics, older adults with dementia should be allowed to stop or cease use of a technology or simply choose not to use it. As one participant suggested, “Alternative options that mean these do not need to be implemented for people/care dyads who just don't want them. People do have different preferences, and they vary, so there should be alternative ways to care for people if they are not comfortable doing these.”

#### Restrictions for Data Security and Unsanctioned Third-Party Use

Most participants emphasized the need to make mandatory best practices for data security, including for example, two-factor authentication, encryption, HIPAA compliance, and permissions. All data transmitted within and outside of the home can and should be encrypted, according to a number of participants. Some expressed concern over the possibility that data would be used by insurers to deny coverage or increase premiums. Disclosures should indicate that data won’t result in higher costs. Consumers, they asserted, should have control over their data. Many echoed this participant’s suggestion: “Ensure that enrollment is NOT through a primary care or insurance related benefit so that the data collected is the property of the individual and/or family member.” Others wrote that data should not be used for marketing or any use other than care provision and that if data are used for research to improve care, consent from individuals should be obtained first. Data should be shared on an opt-in basis. Participants wanted assurances of data privacy and to know how that will be guaranteed, noting that limiting access to data should be carefully controlled.

#### Require Data Transparency

Closely related to the issue of data security is that of data transparency. Requiring data transparency means addressing the following questions: Where are the data going? Who can use them? What are the algorithms used to trigger intervention? Are the data being monetized? In addition to transparency about the answers to these questions, devices should actively provide reminders of how they operate, who is controlling them, and how data are being collected and used. The design concept of feedback should be enabled to alert people when monitoring is occurring; this might be in the form of making a noise when it’s turning on or using visual feedback like the colored LED ring on Alexa that indicates when the device is listening. Transparency and accessible communication at each of these levels is important to mitigate some of the identified risks.

#### Enable Informed Consent

One component of this transparency of data usage is ensuring that consent for using the technology is given by older adults and making sure that consent is fully informed. One participant explained:

Overall, I think for me the most important consideration in cases where there is no control over how the technology was developed, is a solid, ongoing and fully informed consent process, so any way to aid this is valuable: values assessment, maybe multimedia tools to make sure end-users understand what happens to their data/who it's shared with/etc., clear illustrations of possible benefits and harms, etc.

Another participant wrote that while achieving consent may not always be possible, “all of it needs to be with consent.” This can present an ethical conflict, as illustrated in this comment about virtual reality: “Although VR might not seem ethically problematic, the degree of immersiveness might be problematic for people with dementia if it is put on them without them having a viable way to say they do not wish to be a part of it.” Another wrote that “monitoring related to security (entry and leaving, audio surveillance, video surveillance, etc.) brings with it an increased likelihood of illicit use by unscrupulous persons, which may include family. Elders need to be well counseled in the risks associated with monitoring even if it's for their own good.”

Participants suggested establishing informed consent as a process rather than a one-time event so that people could revisit their use. They recommended repetitive opportunities to try it out, decline to use, and then try again as technologies change and develop. Some acknowledged the possibility that a person living with dementia would not have static preferences as their condition progresses and thus recommended that values assessment be done at earlier stages of disease progression. Conversations that included advanced directives about the use of technologies that monitor and use AI were called for. Finally, participants suggested that when a device has been chosen, people living with dementia could be included in the installation process and onboarding.

Table 5 shows the technologies most commonly selected in response to the following prompt: “Consider which of the below are the most likely to be threatening to values that matter to older adults. Please select six that most warrant a conversation with an older patient when families are deciding about using them in their care.”

**Table 5:** Number of participants selecting technologies that most warrant a conversation about potential use

Number of participants out of 21	
18	Recording audio in a person’s home to respond to emergencies and security threats, monitor socialization, or detect cognitive change and other conditions
16	Video conferencing that would allow a caregiver to turn the webcam on and “enter” the room visually for social connection and visual assessment of person and home
15	Location tracking within the home to detect variation in patterns of activity
13	Location tracking outside of the home to monitor safety and social activity
12	Using an AI virtual agent or socially assistive robot for non-human companionship to ease loneliness and prompt cognitive or physical engagement
11	Detecting movements through the apartment door to confirm return home and monitor outdoor activity frequency or presence of workers, meal delivery, and visitors
11	Remotely monitoring physiological variables for frequent assessment to predict and manage health-related risks

#### Proactively Educate Users

Participants described the need for companies to be responsible for educating consumers about their devices, suggesting that this should be done using language that emphasized person-centered care practices. Participants recommended including disclosures explaining that all systems can fail or have glitches, that oversight and backup provisions are necessary, and that AI is not a substitute for contact with a person living with dementia. User manuals could proactively disclose potential concerns and how to adapt systems, such as reducing frequency of non-critical alerts. A participant noted that it should be acknowledged that “caring” for the technology represents additional work for caregivers and that some of the data gathered may need clinician interpretation before release to the family.

Another participant offered a suggestion for vendors to help families have conversations and plans for using technology:

A service wrap-around to products that include any or all of the following: examples of how the technology has been used by others, conversation suggestions to support family discussions around the use of the technology, access to human support for the conversation. Human support could take the form of a vender-employed care guide or connection to peer experts in the users' community.

Others recommended educational materials to guide caregiver interactions. For products that have significant potential effect on quality of life, care, routine, or privacy, one suggested mandatory onboarding materials or training that the caregiver should complete before using the product.

## Discussion of Implications

Drawing on the expertise of 21 gerontechnology academic and industry domain experts, we identify which technologies may be the most prevalent in dementia care in five years, along with their individual and shared potential benefits and risks. This article offers a number of unique contributions. These include descriptions of dominant technology categories and a detailed list of primary risks and benefits associated with each (Table 2). We describe the range of options offered to help mitigate the risks and to promote person-centered technology use with this growing population.

While support for people living with dementia and families were top of mind for all participants, these findings suggest important implications for all stakeholders, including systems designers, clinicians, ethicists, researchers, and policy makers. Participants explained that the identified risks specific to each technology (Table 2) and the aggregated risks (Table 3) require intervention at multiple levels because they are consequences of choices made at each stage. Some are issues of design that could be mitigated by making different design choices or designing in new options for the user, while others, such as security, may best be addressed through regulatory choices. Intervention should thus begin with systems design and weave into implementation and regulation, following the seven categories described in detail above (summarized in Table 4). Below, we build on the statements provided by participants and discuss further the implications of a subset of their suggestions: employing appropriate design frameworks, enabling control and informed consent, and protecting data and transparency.

### Employing Appropriate Design Frameworks

Many of the concerns raised do need to inform design, and existing frameworks—value sensitive design, privacy-by-design, and the ethical adoption framework—should be incorporated into design processes now. Value sensitive design (VSD) focuses systematically on human values through theory, methods, and practice in the design process (Friedman and Hendry 2019, 3–4). It acknowledges that values cannot be easily isolated, because they sit in relationship with other values (e.g., autonomy and identity, privacy and trust) (Friedman and Hendry 2019). A respondent echoed this fact, writing that “human ‘values’ tend to be mixed up and contradictory.” Friedman and Hendry (2019) use the term “value tensions,” which, they write, “conveys the idea of values potentially in opposition but allows for solutions that balance each value in relation to the others, such that the adjudication of the tension holds each value intact” (p. 44). This redirects design away from zero-sum thinking. Value tensions can exist internally, between people, and among institutions.

Attention to human values in design is still a novel approach, and its lack of mainstream adoption has negative consequences for products and the people who use them (Davis 2015). If values and potential value tensions were unearthed in the design process, clinicians, care coordinators, and families could be spared the burden of having to navigate products that aren’t grounded in values. Value sensitive design has promise to lead to creative accommodations in thoughtfully designed technologies that are made with human values at the center.

The privacy-by-design framework was developed in recognition of the increasing value of data and the urgency to manage them responsibly (Cavoukian 2011). Among its seven foundational principles is making user-centric choices, such as offering strong privacy defaults, transparency, and appropriate notice, and empowering user-friendly options. The privacy-by-design approach is embedded and proactive, anticipating and preventing privacy invasion. It positions privacy as the default such that users do not need to take action to protect it (Cavoukian 2011). It eschews the unnecessary trade-offs of false dichotomies (e.g., privacy vs. security) in favor of accommodating positive-sum interests (Cavoukian and Emam 2010; Cavoukian 2011). Intimate privacy threats have perhaps received greater attention where they occur in intimate partner and parent/child relationships. Schneier and Levy (2020) include elder care in their analysis of privacy threats in intimate relationships. They remind readers, “The fact that intimate information-sharing is widespread and often accepted should not lead us to be unreflective about very real privacy threats within intimate relationships. Rather, it makes it all the more important to consider how intimate privacy threats occur, when they are unwelcome, and how to reason about them conceptually” (Schneier and Levy 2020, pp. 2–3). Threats to privacy was among the most commonly endorsed risks of using these dementia care technologies, which indicates that there is significant work to be done yet to protect the privacy of individuals and families.

Finally, the ethical adoption framework is grounded in bioethics and was developed specifically for technology for dementia care. It addresses not just the design but also the integration of design, development, deployment, and use (Robillard et al. 2018). The framework includes 18 recommendations based on five pillars that are supported by empirical evidence: inclusive participatory design, emotional alignment, adoption modelling, ethical standards assessment, and education and training (Robillard et al. 2018). This framework targets multiple phases of technology trajectory, including early design, delivery, adoption promotion, ethical standards review, user testing, launch, and continued use (Robillard et al. 2018).

### Enabling Control and Informed Consent

Enabling informed consent and proactively educating users were prioritized needs, as were built-in options for choice and control. Participants cautioned against generalizing ideas of how people prioritize implicated values, stressing the need to understand that this will vary by person. This work and others (Mulvenna et al. 2017; Berridge 2018; Wangmo et al. 2019; Berridge and Wetle 2019) suggest that we need to further investigate how to support personalized patient and family decision-making about these forms of technologies used in dementia care with attention to alignment with participants’ cultural values (Berridge et al. 2019).

To a great extent, those technologies participants rated most likely to cause family conflict were also predicted to be most prevalent in five years in dementia care. Because these are predicted to be the most prevalent soon, the need to learn how to ensure that families are well counseled about their risks may be the most immediate. The five deemed most likely to call for conversations and predicted to be among the most prevalent were video conferencing that allows a caregiver to turn the webcam on and “enter” the room; location tracking outside of the home; location tracking within the home; detecting movements through an apartment door; and remotely monitoring physiological variables. The technology most likely to cause conflict—recording audio—was not expected to be as prevalent as the other options, though some of those do include audio recording capacity.

Using AI conversational agents was ranked fifth for potential to cause conflict and seventh for prevalence in five years. A number of respondents used the word “problematic” in reference to using AI for companionship and social interaction. The primary ethical issues with artificial companions discussed in the literature were also noted by participants. These include the likelihood of deception, privacy invasion through monitoring and tracking, and the possibility that such devices could ease or contribute to social isolation (Robillard et al. 2020; Wangmo et al. 2019). The potential for artificial companions to ease loneliness is actively designed for and promoted, yet the regulatory landscape is weak (Portacolone et al. 2020), uninformed by input from older adults and advocates (Robillard et al. 2020). The current study’s findings align with the recommendation that resources are needed to examine the ethical aspects and implications of such use—research that meaningfully engages older adults and caregivers—and to put protective policies in place prior to broader implementation (Robillard et al. 2019).

Strikingly little research has focused on the convergence of policy and ethical issues in dementia care technology use (Robillard et al. 2019). A recent policy analysis describes state Medicaid waiver programs’ difficulty regulating geo-tracking, activity sensors, and web-cameras that some are allowing and paying for. Program managers report struggling to understand which circumstances warrant use of these devices and how to support ethical decision making for beneficiaries with cognitive impairment (Berridge 2018). Another ethics analysis of 23 policy documents from four Alzhiemer’s Associations in four countries found that they prioritize benefits of technologies while inadequately discussing potential harms. The authors note “a lack of formal guidance and governing for both existing and emerging technologies” (Robillard et al. 2019). The current study further underscores why—absent such guidance and regulation—families, front line staff, and policy makers will continue to struggle with ethical and low-risk use of dementia care technology.

### Protecting Data and Transparency

Data security and transparency were also strong themes. These data collected about older adults are potentially financially valuable and present risks to personal privacy. Consumer protections, enacted through policy or law, that mandate best practices such as two-factor authentication, encryption, HIPAA compliance, data-use transparency, and consumer controls (e.g., clearly-communicated opt-in rather than opt-out options) will be essential.

Data transferred from a range of devices—particularly those that collect physiological data—might be used by insurance companies to restrict access or increase costs of care. Preventing direct relationships between health insurers and device companies was recommended. Regulation appears overdue. Health insurance companies such as Aetna are launching products that use data collected by personal data devices—in this case, a collaboration with Apple to launch an app using Apple Watch data (Shieber 2019). The public also has good reason not to trust that its data are safe from unsanctioned use, as shown recently by the secret transfer by Ascension of nearly 50 million consumers’ medical data to Google (Project Nightingale) (Pilkington 2019). Study participants felt that family caregivers should be relieved of the burden of such risks. This will require significant policy work and new mechanisms for regulators to keep up with device changes (Duggal et al. 2018).

The specific recommendations offered here are relevant to designers, clinicians, ethicists, researchers, and policy makers. It is clear that there is no catch-all solution and that intentional choices need to be made to address these risks across design and implementation.

### Limitations

A greater proportion of academic researchers than industry representatives participated in this Delphi process; however, many of them were also engaged in product development as part of their research. The majority of participants did not have specific expertise in ethics, and participants were not asked to comment on ethical issues; rather, questions were framed in terms of potential benefits and risks. While ethical issues were raised, this study does not and was not intended to represent a thorough review of ethical issues. For explicit focus on ethics, see for example, Wangmo, et al. (2019) and Vallor (2016). Still needed is the engagement of experts in AI and other applications who are not working in gerontechnology. The methods of convenience and snowball sampling to identify prospective participants may limit the representativeness of our sample.

The focus of this study was on technologies used in dementia care by families. While some insights are broadly applicable, specific contexts of nursing homes or other long-term care residential settings were not explored. Further, this study does not address the problem of the exclusion of people living with dementia in research (Taylor et al. 2012) that is also a limitation in gerontechnology. More studies that include people living with dementia are needed if we are to understand the full scope of potential risks, benefits, risk mitigation efforts, and the ways digital technologies open and close ways of being and experiencing daily life.

## Appendix: List of participants

Walter R. Boot, Professor of Psychology, Florida State University.

Christine Cassel, Presidential Chair, University of California at San Francisco.

Neil Charness, William G. Chase Professor of Psychology and Director, Institute for Successful Longevity, Department of Psychology, Florida State University.

R. Scott Collins, Chief Executive Officer, Link-age.

Kay Connelly, Professor and Associate Dean for Research in the School of Informatics, Computing, and Engineering, Indiana University.

James Fogarty, Professor, Computer Science and Engineering, University of Washington.

Joseph Gaugler, Professor and Robert L. Kane Endowed Chair in Long-Term Care and Aging, School of Public Health, University of Minnesota.

William Kearns, Associate Professor, Meritorious (Retired) Rehabilitation and Mental Health Counseling, Department of Child and Family Studies, University of South Florida, President, International Society for Gerontechnology.

Amanda Lazar, Assistant Professor, College of Information Studies, University of Maryland.

Diane Mahoney, Professor Emerita MGH Institute for Health Professions, School of Nursing.

Barbara Marshall, Professor of Sociology, Trent University.

Debra Oliver, Professor and Paul Revare, MD, Family Professor of Family Medicine, University of Missouri.

Laurie Orlov, Tech industry veteran and founder, Aging in Place Technology Watch.

Carla Perissinotto, Associate Professor in the Division of Geriatrics and Associate Chief for Geriatrics Clinical Programs, University of California San Francisco.

Marilyn Rantz, Curators' Professor Emerita, School of Nursing, University of Missouri, Executive Director, Aging In Place Project and Sinclair Home Care.

Julie Robillard, Assistant Professor of Neurology, University of British Columbia and Associate Director of Neuroethics Canada.

Brian Shulman, Director, Business Development, Intuition Robotics.

Andrew Sixsmith, Professor in the Department of Gerontology, Simon Fraser University, Director of the Science and Technology for Aging Research (STAR) Institute, Scientific Director of AGE-WELL NCE, Canada’s AgingTech network.

Nancy Vuckovic, Director of Experience Design, Cambia Health Solutions.

Erin Washington, Co-Founder and Chief Product Officer, Embodied Labs.

Jutta Williams, Sr. Technical Program Manager, Facebook.
