# Correction: Domain Experts on Dementia-Care Technologies: Mitigating Risk in Design and Implementation

**DOI:** 10.1007/s11948-022-00423-z

**Published:** 2023-01-18

**Authors:** Clara Berridge, George Demiris, Jeffrey Kaye

**Affiliations:** 1 School of Social Work, University of Washington, Seattle, WA USA; 2 School of Nursing and Perelman School of Medicine, University of Pennsylvania, Philadelphia, PA USA; 3 School of Medicine, Oregon Health and Science University, Portland, OR USA

**Correction to: Science and Engineering Ethics (2021) 27:14** 10.1007/s11948-021-00286-w

In the Funding section of this article the grant number relating to the National Institutes of Health given for Jeffrey Kaye was incorrectly given as „U2C-AG0543701 to J.K.“ and should have been „**U2CAG054397** to J.K.“.

The original article has been corrected.

